# A Review of Ecosystem Services Research Focusing on China against the Background of Urbanization

**DOI:** 10.3390/ijerph19148271

**Published:** 2022-07-06

**Authors:** Qindong Fan, Xiaoyu Yang, Chenming Zhang

**Affiliations:** School of Architecture, North China University of Water Resources and Electric Power, Zhengzhou 450046, China; qindongf@ncwu.edu.cn (Q.F.); zhangchenming@ncwu.edu.cn (C.Z.)

**Keywords:** ecosystem services, urbanization, progress, China, international

## Abstract

The change in landscape patterns caused by urbanization is one of the main reasons for the degradation of global ecosystem services. Reducing the negative impact of rapid urbanization on ecosystems and promoting the coordinated development of cities and ecosystems have become a hot topic around the world. Based on Web of Science Core Collection and CNKI database papers, this study conducts a bibliometric analysis of ecosystem services research against the background of global urbanization from 2000 to 2022. At the same time, the research hot spots, regional distribution, research trends, and research contents are summarized by taking China as the key research area. The results show that: (1) the research hot spots of ecosystem services against the background of urbanization are generally the same in China and the world. Both of them are based on landscape pattern or land use; the research scale is from macro to micro; and the research method is from static to dynamic. (2) From the perspective of ecosystem service types, the four types of ecosystem service have been studied in China and other parts of the world, but there are differences in the specific types, quantity, and regional distribution. (3) Whether in China or other regions of the world, the studies on the trade-offs of ecosystem services against the background of urbanization are mainly at medium and large scales. Finally, ecosystem service bundles research, systematic thinking, and the combination of ecosystem services and territorial spatial planning against the background of urbanization are pointed out as key aspects of future research.

## 1. Introduction

Cities can not only gather resources, facilitate transportation, and provide services for residents, but they also promote economic, social, and cultural development [1,2]. Urbanization is a process in which rural and surrounding natural land is transformed into urban land [3]. Ecosystems exist on surface land [4]. The land use change in the process of urbanization affects the types, areas, and spatial distributions of regional ecosystems [5], resulting in changes in the structure, process, and function of the ecosystems, along with changes in the services supplied by the ecosystems. Ecosystem services are the various benefits that humans obtain directly or indirectly from nature [6]. Ecosystem services include tangible products (such as grains, vegetables, raw materials, energy, etc.) and intangible products (such as climate regulation, water conservation, soil conservation, air purification, recreation, etc.) [7]. Ecosystem services are the foundation of human well-being. The degradation of ecosystem services caused by urbanization has threatened human well-being [8]. Therefore, it is of great significance for scholars to carry out research on ecosystem services against the background of urbanization from a global perspective [9]. Currently, urbanization mainly takes place in developing countries [10]. As a representative of developing countries, China’s urbanization process is rapid [11]. Ecosystems in most parts of China are under increasing urbanization pressure, and there is a contradiction between the limited supply of ecosystem services and the huge population demand [12]. China is a typical case of global ecosystem services research. Selecting China and other parts of the world for research can not only help to understand the current status of ecosystem services research against the background of urbanization but also to predict future development trends.

Currently, research on ecosystem services includes studies on ecosystem services assessment [13,14], change [15,16], simulation [17,18], trade-offs [19,20], and management suggestions [9,21], all against the background of urbanization. However, research on ecosystem services regarding urbanization lacks systematic integration and comparative analysis, as the research results are mostly based on individual case studies. For this paper, we used Web of Science Core Collection and CNKI database papers from January 2000 to January 2022 as data sources and analyzed “hot spots”, regional distribution, and the content of ecosystem services research against the background of urbanization from both Chinese and international (including China) perspectives using a bibliometric analysis method.

In addition, we have highlighted existing problems and future development directions for current research in order to provide a reference for theoretical research and applied management of ecosystem services against the background of urbanization.

## 2. Research Progress of Ecosystem Services against the Background of Urbanization

### 2.1. Research Hot Spots

**Figure 1:**
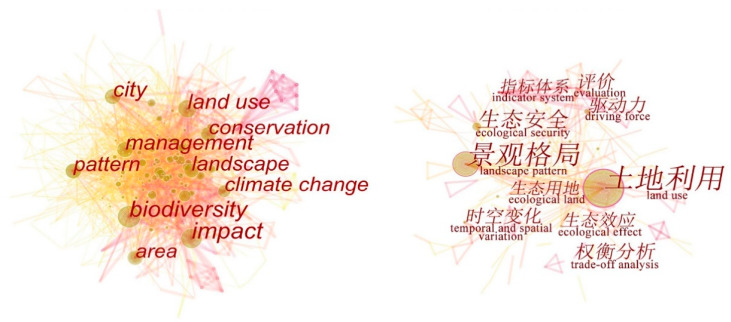
Co-occurrence of international keywords (**left**) and domestic keywords (**right**).

Keywords such as “urbanization” and “ecosystem services” were searched in the Web of Science Core Collection and CNKI database with 2599 English-language and 760 Chinese-language papers obtained after removing duplicate papers. Next, a keyword co-occurrence analysis was conducted to extract the top 10 keywords in the number of studies (Figure 1).

The change in surface landscape pattern, to which ecosystem services are attached, is one of the most obvious characteristics of urbanization [22]. Landscape pattern refers to the spatial distribution of land types of different sizes, types, and shapes [23]. Because the ecosystem is attached to the landscape, the landscape pattern determines the type, quantity, function, and distribution of ecosystem services. From the keywords “landscape”, “pattern”, and “land use” in Figure 1, it can be seen that landscape pattern or land use change against the background of urbanization is a common hot spot in the study of ecosystem services at home and abroad. In general, domestic research has focused on the impact of abiotic factors on ecosystems during the process of urbanization. China also pays attention to the measurement, evaluation, and driving force analysis of ecosystem services against the background of urbanization. In contrast, global research focuses more on ecosystem services at different scales and the impact of urbanization on biodiversity and climate. In addition, more papers focus on the topic of ecosystem service conservation and management.

### 2.2. Distribution of Studies

**Figure 2:**
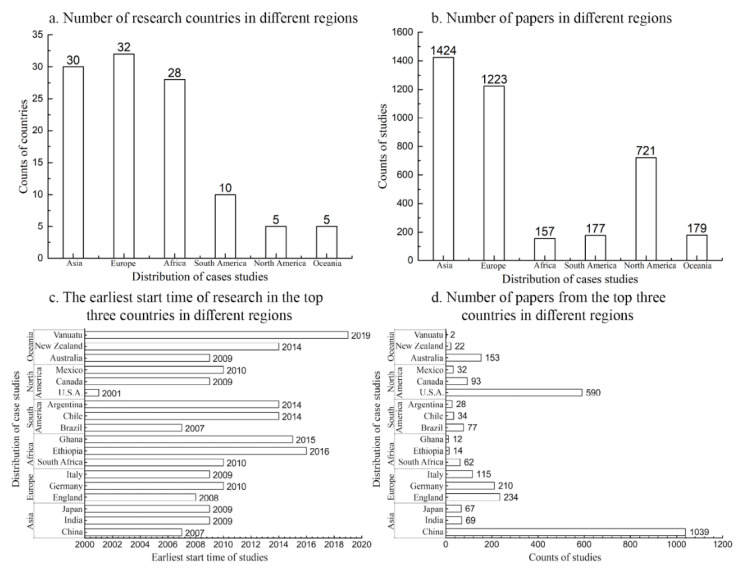
Study areas and paper distribution.

The retrieval results from the Web of Science Core Collection were classified according to different regions, and ArcGIS was used to draw distribution maps of the research region (Figure 2).

Asia, Europe, and North America have a large number of papers and relatively comprehensive research content, and their research models are relatively mature from a global perspective (Figure 2a,b). Most developing countries in Africa and South America have relatively few studies and relatively scattered content due to the slower urbanization process and limited scientific research level [24,25]. However, these countries will be the main areas of urbanization in the future, and their changes will have a greater impact on the global environment. There is a clear need, therefore, for more extensive and high-level research in these areas.

From a regional perspective (Figure 2d), there are also differences between studies in different countries within the same continent, especially in Asia, North America, and Oceania. China has the largest number of studies in Asia; the United States has the largest number of studies in North America.

For example, China, America, Germany, Australia, and Britain have published a large number of papers from a national perspective (Figure 2c,d). China has published the largest number of studies, which may be due to its rapid and large-scale urbanization and the prominent contradiction between ecology and urbanization [13]. Alternatively, it may be because of the scientific research level of ecology and geography, which has improved significantly in recent years [26,27]. Simultaneously, the number of high-level papers published in the United States is relatively large (high-level papers refer to papers in journals falling into the highest quartile (Q1) in their category and are among the top 25% in the impact factor distribution), due to the early start of research in geography and ecology.

### 2.3. Research Trends

**Figure 3:**
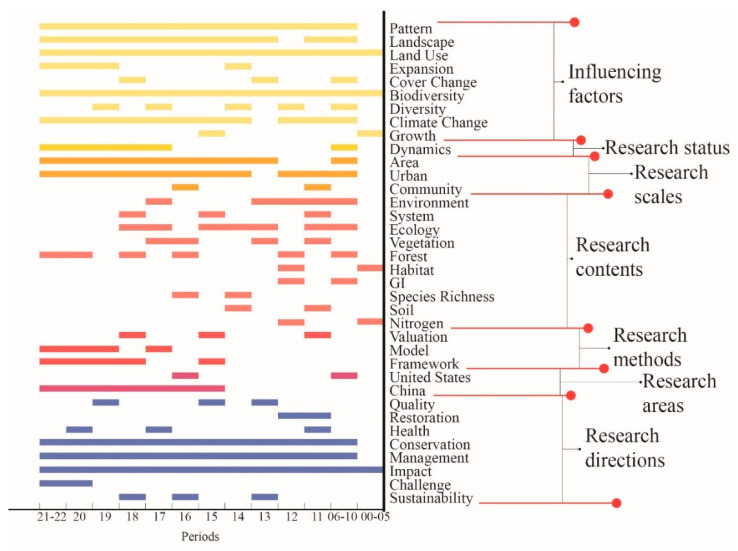
Changes in study trends.

A keyword co-occurrence analysis was conducted on the papers retrieved from the Web of Science, and keywords were arranged on the axis of time, as shown in Figure 3 below.

The following study trends were observed.

(1) Analysis of influencing factors: “Landscape”, “Pattern”, “Land Use”, and “Biodiversity” were analyzed throughout the study period. (2) It can be seen from the research status that, with the deepening of research, static quantitative evaluation gradually decreases, and dynamic simulation and prediction research gradually increases. The reasons are as follows. Static research can only analyze the state of ecosystem services at the current moment or at a certain moment in the process of urbanization. However, urbanization is a dynamic process, and land use is constantly changing, resulting in a constantly changing supply of ecosystem services. Dynamic simulation and prediction of ecosystem services against the background of urbanization is more realistic, enabling stakeholders to understand future scenarios and develop response strategies in advance [28]. (3) Research scales are increasingly diversified, such as from “Uban”,”Area” to “Community”. (4) Research content gradually deepened and became more detailed, for example, from overall urbanization to “Green Infrastructure”, from macro “Habitat” to specific “Species Richness”, from ecosystem service index evaluation to specific mechanism factors analyses of water, nitrogen, soil, and carbon. (5) Model research is gradually increasing, and research methods are more and more generalizable. It can be seen from the figure that the keywords “Model” and “Framework” have been used more since 2018, and research on the keyword “Evaluation” has decreased relatively. This is because most model studies involve the change mechanism of ecosystem services, which can reflect changes in ecosystem services more scientifically [29]. Ecosystem service assessment extends to model studies, multi-scenario simulations, and multi-objective optimization. The advantage of multi-scenario simulation and multi-objective optimization is that they can assume future land use scenarios or set the supply target of ecosystem services in advance and then formulate land management policies in a targeted manner. This method provides a variety of references and options for managers or decision-makers, which is beneficial for scientifically formulating land management plans [30].

(6) Analyzed from the study area: “China” appeared as a keyword in an increasing number of studies, while “United States” as a keyword gradually decreased. (7) From the trend analysis, the interaction between urbanization and ecosystem services and the protection and management of ecosystem services against the background of urbanization have become current and future research topics. The reasons are as follows. Urbanization is currently the most important driving force of global land use change [31]. In addition, ecosystem services depend on land for their existence. Therefore, changes in land use during urbanization can lead to changes in the supply of ecosystem services. Finally, the supply of ecosystem services has important implications for human well-being. Therefore, it is very important to protect and manage ecosystem services against the background of urbanization.

## 3. Research on Different Types of Ecosystem Service against the Background of Urbanization

According to MA [6], ecosystem services are divided into provisioning services (such as food production and water supply, etc.), regulating services (such as flood regulation and climate regulation, etc.), supporting services (such as habitat quality and soil conservation, etc.), and cultural services (such as recreation and tourism, etc.).

### 3.1. The International Perspective

**Figure 4:**
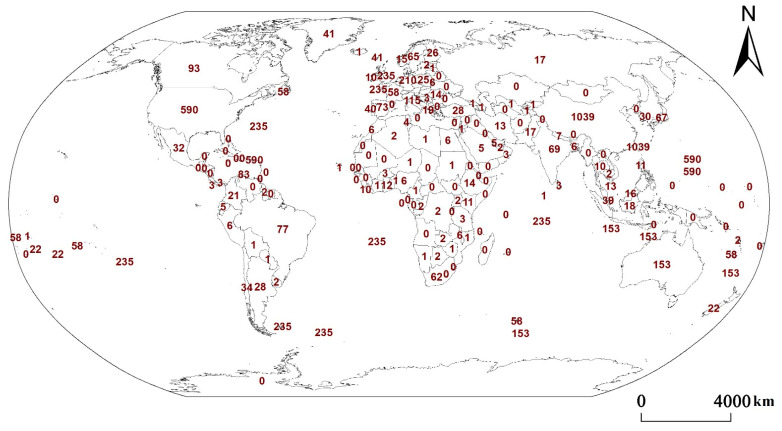
Quantity distribution of international ecosystem services papers. The numbers in the figure are the number of research papers in each region.

**Figure 5:**
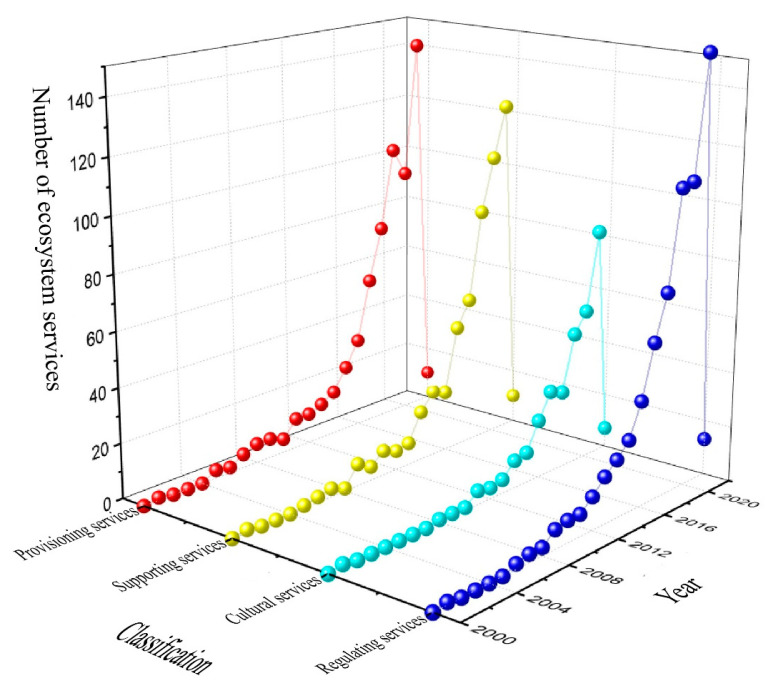
Studies on different types of ecosystem services.

In order to study the impact of urbanization on different types of ecosystem services, the papers retrieved from the Web of Science from 2000 to 2022 were mapped (Figure 4 and Figure 5).

As can be seen from Figure 4, the number of studies on ecosystem services is closely related to the level of urbanization. Countries with more than 50 papers, such as the United States, the United Kingdom, Germany, Australia, Italy, Canada, the Netherlands, Spain, Japan, Sweden, France, and Switzerland, are concentrated in Europe, North America, and Oceania. They are all developed countries with high levels of urbanization. Other countries, such as China, Brazil, India, and South Africa, are located in Asia, Africa, and South America. They are relatively developed countries in developing countries with a relatively high level of urbanization. The countries with zero studies are mainly distributed in Asia, Africa, and South America, and most of these countries are developing countries with low levels of urbanization. Moreover, the countries with the largest number of studies are China and the United States. The most studied country in Africa is South Africa, and the most studied country in South America is Brazil.

There are more cultural services in North America, Europe, and Oceania and more provisioning services in Asia, Africa, and South America [24]. Regulating and supporting services are broadly studied across continents but vary slightly in specific types [32]. There are more studies on pollination in North America, Europe, and Oceania but less in Asia, Africa, and South America [33].

The amount of research on the four types of service has shown a general upward trend and has increased markedly since 2010 against the background of urbanization (Figure 5). The number of papers on regulating services (31%), provisioning services (29%), and supporting services (25%) is greater than that of cultural services (15%), from the analysis of paper quantity. In terms of research time, research on cultural services is relatively behind the other three services. The reason for the significant decline in the four types of service for 2022 is that paper selection ends in January 2022.

Due to the heterogeneity of the geographical landscape and people’s values and lifestyle preferences, ecosystem services research against the background of urbanization is mostly based on individual case studies. However, on the whole, with the advancement of urbanization, urban agriculture has gradually emerged, and traditional food production and other provisioning services are gradually moving spatially towards the urban fringe [34]. At the same time, with improvements in the ecological awareness of decision-makers, green infrastructure in urban areas is gradually increasing.

However, due to the decrease in natural habitats and the increase in impervious surfaces [35,36], regulating and supporting services are in decline. In addition, most cultural services have experienced destruction and reconstruction in the urbanization process; on the one hand, changes in land use have led to a decrease in cultural services, while on the other hand, the construction of green parks has increased cultural services [35,37,38].

### 3.2. China’s Perspective

**Figure 6:**
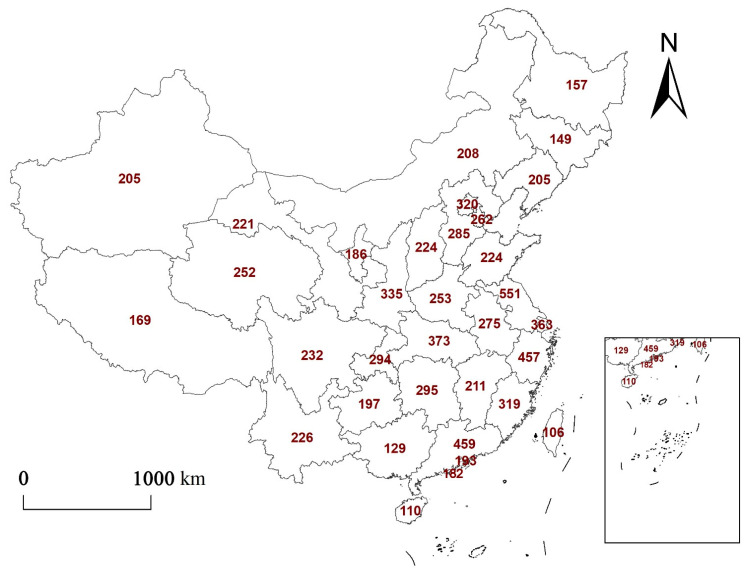
Distribution of studies on ecosystem services. The numbers in the figure are the number of ecosystem services studies in each region.

**Figure 7:**
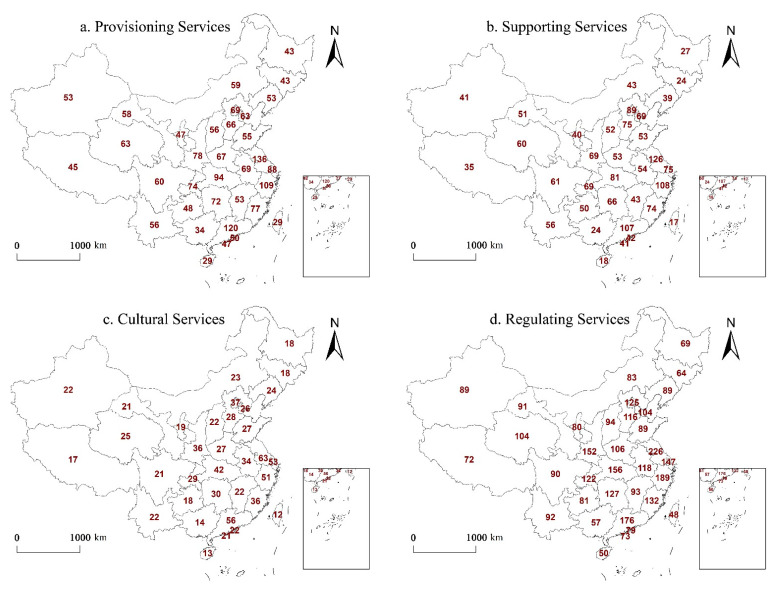
Distribution of studies on different types of ecosystem service. The numbers in the figure are the number of different ecosystem services studies in each region.

Papers with the keywords “urbanization”, “ecosystem service”, and “China” were retrieved from the Web of Science Core Collection and CNKI database The distribution of the research areas was mapped using ArcGIS10.2, as shown in Figure 6 and Figure 7.

As shown in Figure 6, studies on ecosystem services against the background of urbanization show clear regional differences. In general terms, however, there is a synergistic relationship between the number of studies on ecosystem services and the level of urbanization. For example, (1) regions that have high levels of urbanization [39], such as Jiangsu, Zhejiang, and Guangdong, have more studies (more than 400 papers) than other provinces. (2) Beijing, Hubei, Shaanxi, and other regions with a strong scientific research background and relatively high level of urbanization [40] have published more research papers than Xizang, Jiangxi, and Guangxi provinces. (3) There are also fewer studies conducted in western and northeastern China than in the central and eastern coastal regions, particularly in Xizang, Guangxi, Heilongjiang, and Jilin. Most of the studies in these provinces are carried out in conjunction with the surrounding areas.

Figure 7 shows that the number of studies on regulating services (41%) is significantly greater than the other services (23% supporting services, 25% provisioning services, 11% cultural services). This may be because China’s rapid urbanization over the past few decades has led to a significant decline in environmental quality and frequent natural disasters, such as haze [41], urban waterlogging [42], and the heat island effect [43], which has led scholars to focus more on regulating services that have a direct impact on the environment.

Figure 7a,b shows that the distribution locations and research quantity of provisioning and supporting services in China are relatively similar. These services are mostly distributed in central and eastern regions, while provisioning services, such as food production and raw material production, are always accompanied by the consumption of water resources, which inevitably leads to a decline in soil formation and protection, biodiversity, and other supporting services [44]. However, the urbanization process in the western and northeastern regions is comparatively slow, and the changes in provisioning and supporting services are relatively small, meaning that corresponding research is relatively scarce.

Cultural services are closely related to the level of urbanization in the region (Figure 7c) [45,46]. The municipalities directly under the central government and the eastern coastal areas with a higher level of urbanization have more research on cultural services. On the whole, there are more studies on regulating services than on other services (Figure 7d), with only a few studies in Guizhou.

### 3.3. Similarities and Differences between Chinese and International Studies

In terms of the number of studies, generally speaking, there are more studies in areas with relatively high levels of urbanization. There are relatively more studies on regulating services in all regions, and relatively few studies on cultural services. However, the number of studies on regulating services in China is far greater than that on provisioning and supporting services. In terms of research distribution, there is little research on provisioning services and more research on cultural services in developed countries internationally, but there are more provisioning services and cultural services in China in areas with a high level of urbanization. In terms of research content, there is much international and Chinese research on supporting and regulating services, but the international focus is more on pollination services in supporting services, especially in countries in Europe and North America.

## 4. Study on Ecosystem Service Trade-Offs against the Background of Urbanization

Ecosystem services do not exist alone in nature, and changes in one service usually affect other services [47]. For example, changes in food production services may lead to changes in services such as soil conservation, carbon storage, and recreation. This complex relationship between ecosystem services is trade-offs [48]. Changes in land use types during urbanization lead to changes in the relationship between services such as biodiversity, climate regulation, and recreation. Trade-off studies are beneficial to understanding the relationship between multiple ecosystem services and reducing the conflict between socioeconomic development and ecological protection [24].

### 4.1. The International Perspective

**Figure 8:**
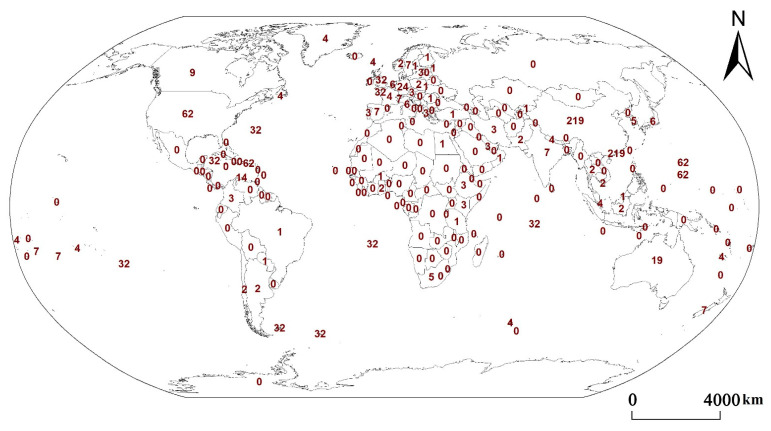
The distribution of the number of international trade-off studies. The numbers in the figure are the number of trade-off studies in each region.

**Figure 9:**
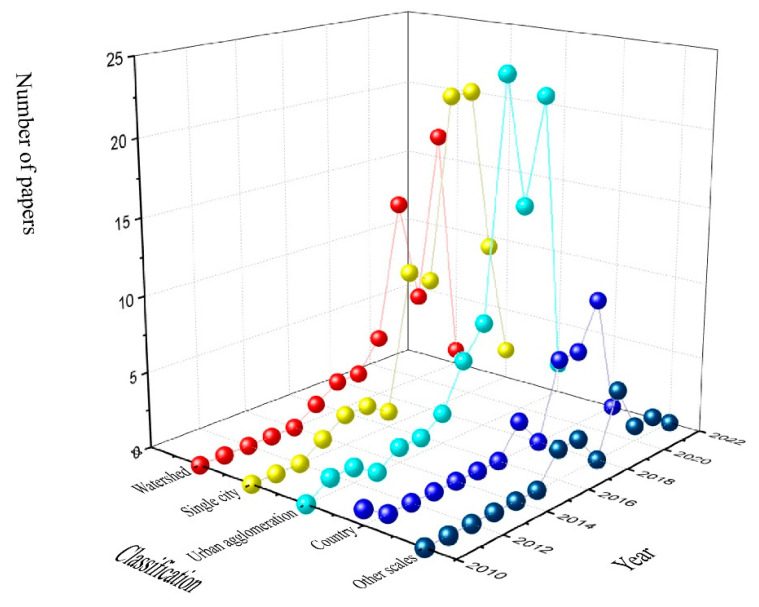
Trade-off studies at different scales.

Trade-offs caused by urbanization have replaced natural competition and have become the dominant factor in the relationship between ecosystem services. Trade-offs have also become the focus of ecosystem services research against the background of urbanization [49,50]. Papers containing “urbanization”, “ecosystem service”, “trade-off”, and other related keywords were retrieved from the Web of Science Core Collection and mapped (Figure 8 and Figure 9).

At present, there are only a few studies on trade-offs. As can be seen from Figure 8, there are generally more studies on trade-offs of ecosystem services in areas with a higher level of urbanization. Among the countries with more than 10 papers, the United States, the United Kingdom, Germany, Australia, and the Netherlands are concentrated in Europe, North America, and Oceania, and they are all developed countries with a high level of urbanization. Only one developing country in Asia, China, has more research. Most of the countries with zero studies are located in Asia, Africa, and South America, and most of these countries are developing countries with low levels of urbanization.

It can be seen from Figure 9 that research on trade-offs of ecosystem services against the background of international urbanization is on the rise. The research started around 2010 and gradually increased after 2015. The research scale has developed from countries and urban agglomerations to the coexistence of multi-scale studies including watersheds and single cities.

The overall research is mainly on medium and large scales, while there are only a few studies on trade-offs between large scale (global, multinational) and small scale (urban green spaces). The decline in the number of studies at all scales in 2022 is due to the fact that papers were only included up to January 2022.

### 4.2. China’s Perspective

**Figure 10:**
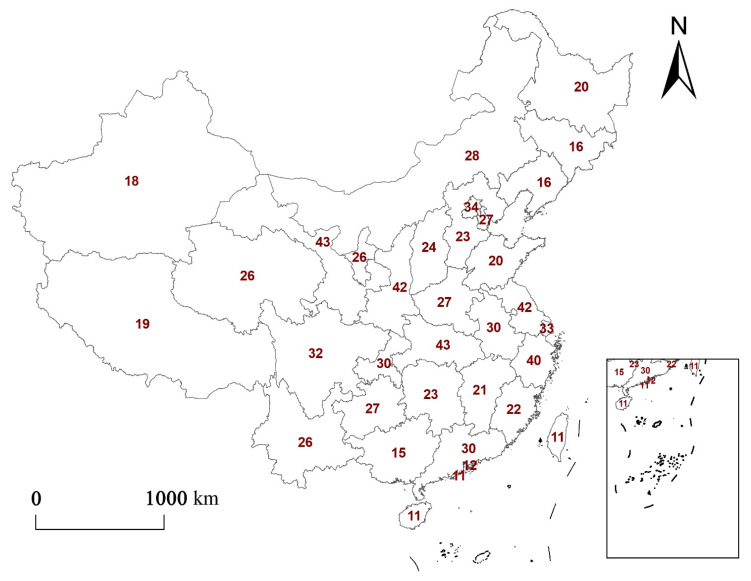
The distribution of the number of international trade-off studies. The numbers in the figure are the number of trade-off studies in each region.

The topics of “urbanization”, “ecosystem services”, “trade-offs”, and “China” were retrieved from the Web of Science Core Collection and CNKI database The number of papers on trade-offs was combined with the regional distribution of provinces and cities in China. The software program ArcGIS was used for mapping (Figure 10).

Figure 10 shows that studies on ecosystem service trade-offs against the background of urbanization show obvious regional differences. Overall, there are more studies on the trade-off of ecosystem services in areas with high levels of urbanization and their surrounding areas (more than 40 papers). [51]. This may be because studies on trade-offs tend to be medium- and large-scale studies, and watershed- and urban agglomeration-scale studies account for the majority of trade-off studies (53%). When conducting research, relevant scholars mostly choose medium and large scales, and the research area includes areas with high urbanization levels and their surrounding areas.

### 4.3. Similarities and Differences between Chinese and International Studies

Generally speaking, the number of international and Chinese trade-off studies is relatively small; the scales are mostly concentrated on medium and large scales, and the research methods are mostly mathematical statistical methods (correlation analysis and cluster analysis, etc.), model simulation, and multi-criteria analysis [52]. Research on trade-offs between different services not only exists between cultural services and provisioning services (such as the trade-off between urban fringe food supply and urban citizens’ leisure and entertainment) but also between provisioning services and regulating services (such as timber production and carbon storage in urban parks). There are also trade-offs within similar services, such as an increase in food production services on the urban fringe leading to a decrease in other provisioning services. In terms of research content, there are differences between the world and China. Trade-off studies in China are usually trade-offs of ecosystem services at the level of entire cities or urban agglomerations [53,54,55]. International research, particularly in developed countries, is more in-depth, involving more specific landscape types, such as the trade-off of ecosystem services in forests [56] during urbanization.

## 5. The Relationship between Urbanization and Ecosystem Services

There is an interaction relationship between urbanization and ecosystem services; on the one hand, land use changes in the process of urbanization affect the type, area, and spatial distribution of regional ecosystems, resulting in changes in the structure, process, and function of ecosystems. The supply of system services also changes [57,58,59]. On the other hand, due to the limited value of ecosystem services, the ecological environment will affect urbanization through environmental degradation and resource shortage. For example, the decline in food production services will cause food shortages and limited human survival, thus restricting the development of urbanization [60,61]. Clarifying the interactive relationships between urbanization and ecosystem services and strengthening the research relationship between urbanization and ecosystem services will contribute significantly to revealing the relationship between their specific connotations and the realization of refined ecological management and urban development control [62]. At present, studies regarding the relationship between urbanization and ecosystem services usually adopt coupling or decoupling methods, as shown in Table 1. Coupling studies mostly adopt the Coupling Coordination Degree [63], the Dynamic Coupling Degree [64], the Structural Equation Model [65], and other indicator models [66,67,68] to quantify the interactive stress relationship between urbanization and ecosystem services. Index models such as the Tapio model [69] and OECD decoupling index [70] are used to quantify the decoupling relationship between them.

There are relatively few papers on relationship studies between urbanization and ecosystem services, and the research area is mainly China [71]. Moreover, there are clear similarities in the selection of urbanization indicators and ecosystem service types. Therefore, future research should focus on strengthening the study of regional differences by combining the heterogeneity of urbanization and ecosystem services. On this basis, cross-regional systematic research should be implemented in future studies [72].

## 6. Conclusions

The research progress of ecosystem services against the background of urbanization in China is relatively similar to that across the world. Research focuses on landscape pattern and land use. Research contents are from macro to micro, from static to dynamic.

The four ecosystem services have been studied all over the world, but there are differences in the types, quantity, and regional distribution of studies. From the analysis of the types of ecosystem service, there are relatively many studies on regulating services in China and internationally. However, the gap between the number of studies on regulating services in China and other services is more obvious; this may be due to China’s rapid urbanization in the past few decades, which has led to a significant decline in environmental quality and the frequent occurrence of natural disasters. From a specific quantitative analysis, there are more studies on ecosystem services in areas with higher urbanization levels. From the analysis of regional distribution, there are more studies on provisioning services in rural areas, while there are more studies on cultural services in cities [25]. In developed countries, there are relatively more studies on cultural services and relatively few studies on provisioning services, and the opposite is true in developing countries [73].

Ecosystem service trade-off studies against the background of urbanization are mainly at medium and large scales and are mostly concentrated in multiple cities (usually watersheds and urban agglomerations). Trade-off studies in developed countries are more focused on supporting services, regulating services, and cultural services, while research in developing countries is more inclusive of provisioning services.

## 7. Prospects

### 7.1. Study Ecosystem Service Bundles in Depth

Bundles are spatiotemporal aggregations of ecosystem services. Strengthening the study of ecosystem service bundles can more accurately reflect the overall state changes of ecosystem services against the background of urbanization [74], which is helpful to promoting the overall management of ecosystem services. Developing countries, represented by China, need to strengthen local mechanism research of ecosystem service bundles. The rest of the world, especially developed countries, needs to strengthen the applied research of ecosystem service bundles on the basis of mechanism research, especially in urban and rural planning practice.

### 7.2. Strengthen the Systematic Research Thinking of Ecosystem Services

Because of differing levels of urban development, the distribution of ecosystem services also has clear levels of heterogeneity. From a global scale, researchers in developed countries should focus on developing countries such as China, because developing countries are the main areas where urbanization takes place. From a national perspective, the selection of ecosystem service research areas needs to weaken the influence of urbanization level and pay more attention to the research of areas with lower urbanization levels; at the urban–rural scale, we hope to achieve a win-win state of ecological protection and economic development through rational urban and rural planning, which is also the significance of urban and rural planning. It is vital that ecosystem service research against the background of urbanization be strengthened with systems thinking in order to promote urban–rural integration, eliminate regional differences, and realize global ecological harmony.

### 7.3. Ecosystem Services and Territorial Spatial Planning against the Background of Urbanization

At present, land use change in the context of urbanization has become the main reason for the degradation of the quantity and quality of global ecosystem services [75]. Territorial spatial planning is an effective means of managing land [76]. Against the background of urbanization, research on ecosystem services and territorial spatial planning has become the core means of protecting human well-being and has been widely carried out in China and the world.

## Figures and Tables

**Table 1:** Research model.

Study Models	Study Methods	Description
Coupling study	Coupling Coordination Degree Model [63]	Describe the degree of interaction between urbanization and ecosystem services. The greater the coupling degree, the more coordinated the urbanization level and ecosystem services, and the more the two systems tend to develop in an orderly fashion.
	Dynamic Coupling Degree Model [64]	
	Structural Equation Model [65]	
	Coupled Human and Nature Cube [66]	
	Coupling Regulator Model [67]	
	Grey Correlation Model [68]	
Decoupling study	Tapio Model [69]	Describe the degree of uncorrelation between urbanization and ecosystem services over time.
	OECD Decoupling Index Model [70]	

## References

[1] Mendoza G.G., Martínez M.L., Lithgow D., Pérez M.O., Simonin P. (2012). Land use change and its effects on the value of ecosystem services along the coast of the Gulf of Mexico. Ecol. Econ..

[2] Tim M.D., Christina C.H., Ka T.B., Tomas C., Eraser A.J., William W.L.C., Sergio R., Beatrice C., Sarah C., Chris S. (2016). Elasticity in ecosystem services: Exploring the variable relationship between ecosystems and human well-being. Ecol. Soc..

[3] Shrestha M., Acharya S.C. (2021). Assessment of historical and future land-use–land-cover changes and their impact on valuation of ecosystem services in Kathmandu Valley, Nepal. Land Degrad. Dev..

[4] Bennett E.M., Peterson G.D., Gordon L.J. (2009). Understanding relationships among multiple ecosystem services. Ecol. Lett..

[5] Ouyang Z., Zheng H., Xiao Y., Polasky S., Liu J., Xu W., Wang Q., Zhang L., Xiao Y., Rao E. (2016). Improvements in ecosystem services from investments in natural capital. Science.

[6] Millennium Ecosystem Assessment (2005). Ecosystems and Human Well-Being.

[7] Daily G.C. (1997). Nature’s Services: Societal Dependence on Natural Ecosystems.

[8] Quintas-Soriano C., Castro A.J., Castro H., García-Llorente M. (2016). Impacts of land use change on ecosystem services and implications for human well-being in Spanish drylands. Land Use Policy.

[9] Cumming G.S., Buerkert A., Hoffmann E.M., Schlecht E., Von Cramon T.S., Tscharntke T. (2014). Implications of agricultural transitions and urbanization for ecosystem services. Nature.

[10] United Nations, Department of Economic and Social Affairs, Population Division (2014). World Urbanization Prospects: The 2014 Revision, Highlights.

[11] Rong F. (2010). Understanding developing country stances on post-2012 climate change negotiations: Comparative analysis of Brazil, China, India, Mexico, and South Africa. Energy Policy.

[12] Peng J., Wang X., Liu Y., Zhao Y., Xu Z., Zhao M., Qiu S., Wu J. (2020). Urbanization impact on the supply-demand budget of ecosystem services: Decoupling analysis. Ecosyst. Serv..

[13] Xiao R., Lin M., Fei X., Li Y., Zhang Z., Meng Q. (2020). Exploring the interactive coercing relationship between urbanization and ecosystem service value in the Shanghai–Hangzhou Bay Metropolitan Region. J. Clean Prod..

[14] Gao J., Li F., Gao H., Zhou C., Zhang X. (2017). The impact of land-use change on water-related ecosystem services: A study of the Guishui River Basin, Beijing, China. J. Clean Prod..

[15] Yang J., Guan Y., Xia J.C., Jin C., Li X. (2018). Spatiotemporal variation characteristics of green space ecosystem service value at urban fringes: A case study on Ganjingzi District in Dalian, China. Sci. Total Environ..

[16] Wang Y., Dai E., Yin L., Ma L. (2018). Land use/land cover change and the effects on ecosystem services in the Hengduan Mountain region, China. Ecosyst. Serv..

[17] Pickard B.R., Van Berkel D., Petrasova A., Meentemeyer R.K. (2017). Forecasts of urbanization scenarios reveal trade-offs between landscape change and ecosystem services. Landsc. Ecol..

[18] Peng J., Liu Y., Tian L. (2018). Integrating ecosystem services trade-offs with paddy land-to-dry land decisions: A scenario approach in Erhai Lake Basin, southwest China. Sci. Total Environ..

[19] Grêt R.A., Altwegg J., Sirén E.A., Van Strien M.J., Weibel B. (2017). Integrating ecosystem services into spatial planning—A spatial decision support tool. Landsc. Urban Plan..

[20] Sun X., Lu Z., Li F., Crittenden J.C. (2018). Analyzing spatio-temporal changes and trade-offs to support the supply of multiple ecosystem services in Beijing, China. Ecol. Indic..

[21] Liang J., Zhong M., Zeng G., Chen G., Hua S., Li X., Yuan Y., Wu H., Gao X. (2017). Risk management for optimal land use planning integrating ecosystem services values: A case study in Changsha, Middle China. Sci. Total Environ..

[22] Dadashpoor H., Azizi P., Moghadasi M. (2019). Land use change, urbanization, and change in landscape pattern in a metropolitan area. Sci. Total Environ..

[23] Lehmkuhl J.F., Ruggiero L.F. (1991). Forest Fragmentation in the Pacific Northwest and Its Potential Effects on Wildlife.

[24] Luederitz C., Brink E., Gralla F., Hermelingmeier V., Meyer M., Niven L., Panzer L., Partelow S., Rau A.L., Sasaki R. (2015). A review of urban ecosystem services: Six key challenges for future research. Ecosyst. Serv..

[25] Lapointe M., Cumming G.S., Gurney G.G. (2019). Comparing ecosystem service preferences between urban and rural dwellers. Bioscience.

[26] Fu B. (2017). Geography: From knowledge, science to decision making support. Acta Geogr. Sin..

[27] Chen L.D., Li X.Z., Fu B.J., Xiao D.N., Zhao W.W. (2014). Development history and future research priorities of landscape ecology in China. Acta Ecol. Sin..

[28] Liao Q., Wang Z., Huang C. (2020). Green infrastructure offset the negative ecological effects of urbanization and storing water in the three gorges reservoir area, China. Int. J. Environ. Res. Public Health.

[29] Li T., Lv Y.H. (2018). A review on the progress of modeling techniques in ecosystem services. Acta Ecol. Sin..

[30] Peng J., Hu X.X., Zhao M.Y., Liu Y.X., Tian L. (2017). Research progress on ecosystem service trade-offs: From cognition to dicision-making. Acta Geogr. Sin..

[31] Bai X.M., Chen J., Shi P.J. (2012). Landscape urbanization and economic growth in China: Positive feedbacks and sustainability dilemmas. Environ. Sci. Technol..

[32] Kang W., Chon J., Kim G. (2020). Urban Ecosystem Services: A Review of the Knowledge Components and Evolution in the 2010s. Sustainability.

[33] Silva J.L.S., de Oliveira M.T.P., Cruz-Neto O., Tabarelli M., Lopes A.V. (2021). Plant–pollinator interactions in urban ecosystems worldwide: A comprehensive review including research funding and policy actions. Ambio.

[34] Eigenbrod F., Bell V.A., Davies H.N., Heinemeyer A., Armsworth P.R., Gaston K.J. (2011). The impact of projected increases in urbanization on ecosystem services. Proc. R. Soc. B-Biol. Sci..

[35] Richards D.R., Law A., Tan C.S.Y., Shaikh S.F., Carrasco L.R., Jaung W., Oh R.R. (2020). Rapid urbanisation in Singapore causes a shift from local provisioning and regulating to cultural ecosystem services use. Ecosyst. Serv..

[36] Ai J., Sun X., Feng L., Li Y., Zhu X. (2015). Analyzing the spatial patterns and drivers of ecosystem services in rapidly urbanizing Taihu Lake Basin of China. Front. Earth Sci..

[37] Bratman G.N., Anderson C.B., Berman M.G., Cochran B., De Vries S., Flanders J., Folke C., Frumkin H., Gross J.J., Hartig T. (2019). Nature and mental health: An ecosystem service perspective. Sci. Adv..

[38] Riechers M., Strack M., Barkmann J., Tscharntke T. (2019). Cultural ecosystem services provided by urban green change along an urban-periurban gradient. Sustainability.

[39] Qi W., Liu S., Zhao M., Liu Z. (2016). China’s different spatial patterns of population growth based on the “Hu Line”. J. Geogr. Sci..

[40] Xu W., Zhou X. (2016). Evaluation mode of provincial science and technology innovation system based on principal component analysis. Sci. Technol. Manag. Res..

[41] Chen S., Chen D. (2018). Air pollution, government regulations and high-quality economic development. Econ. Res. J..

[42] Zhang W., Li S., Shi Z. (2012). Formation causes and coping strategies of urban rainstorm waterlogging in China. J. Nat. Disasters.

[43] Chen A., Sun R., Chen L. (2012). Studies on urban heat island from a landscape pattern view: A review. Acta Ecol. Sin..

[44] Zhang Y., Long H., Tu S., Ge D., Ma L., Wang L. (2019). Spatial identification of land use functions and their tradeoffs/synergies in China: Implications for sustainable land management. Ecol. Indic..

[45] Bolund P., Hunhammar S. (1999). Ecosystem services in urban areas. Ecol. Econ..

[46] Willits F.K., Luloff A.E. (1995). Urban Residents’ Views of Rurality and Contacts with Rural Places. Rural Sociol..

[47] Hao M.Y., Ren Z.Y., Sun Y.J., Zhao S.N. (2017). The dynamic analysis of trade-off and synergy of ecosystem services in the Guan-zhong Basin. Geogr. Res..

[48] Cord A.F., Bartkowski B., Beckmann M., Dittrich A., Hermans-Neumann K., Kaim A., Lienhoop N., Locher-Krause K., Priess J., Schröter-Schlaack C. (2017). Towards systematic analyses of ecosystem service trade-offs and synergies: Main concepts, methods and the road ahead. Ecosyst. Serv..

[49] Wu J., Zhao Y., Yu C., Luo L., Pan Y. (2017). Land management influences trade-offs and the total supply of ecosystem services in alpine grassland in Tibet, China. J. Environ. Manag..

[50] Su S., Wan C., Li J., Jin X., Pi J., Zhang Q., Weng M. (2017). Economic benefit and ecological cost of enlarging tea cultivation in subtropical China: Characterizing the trade-off for policy implications. Land Use Policy.

[51] Chen Y., Cai G. (2021). A review of population migration and urbanization: Inspiration from the seventh National Census. J. Hehai Univ. Philos. Soc. Sci. Ed..

[52] Deng C.X., Zhu D.M., Nie X.D., Liu C.C., Li Z.W., Liu J.Y., Zhang G.Y., Xiao L.H., Zhang Y.T. (2020). Progress of research regarding the trade-offs of ecosystem services. Chin. J. Eco-Agric..

[53] Zheng W., Ke X., Zhou T., Yang B. (2019). Trade-offs between cropland quality and ecosystem services of marginal compensated cropland–A case study in Wuhan, China. Ecol. Indic..

[54] Shen J., Li S., Liang Z., Liu L., Li D., Wu S. (2020). Exploring the heterogeneity and nonlinearity of trade-offs and synergies among ecosystem services bundles in the Beijing-Tianjin-Hebei urban agglomeration. Ecosyst. Serv..

[55] Yang Y., Zheng H., Kong L., Huang B., Xu W., Ouyang Z. (2019). Mapping ecosystem services bundles to detect high-and low-value ecosystem services areas for land use management. J. Clean. Prod..

[56] Delphin S., Escobedo F.J., Abd-Elrahman A., Cropper W.P. (2016). Urbanization as a land use change driver of forest ecosystem services. Land Use Policy.

[57] Yang G., Ge Y., Xue H., Yang W., Shi Y., Peng C., Du Y., Fan X., Ren Y., Chang J. (2015). Using ecosystem service bundles to detect trade-offs and synergies across urban–rural complexes. Landsc. Urban Plan..

[58] Deng C., Liu J., Nie X., Li Z., Liu Y., Xiao H., Hu X., Wang L., Zhang Y., Zhang G. (2021). How trade-offs between ecological construction and urbanization expansion affect ecosystem services. Ecol. Indic..

[59] García N.A., Geijzendorffer I.R., Baró F., Roche P.K., Bondeau A., Cramer W. (2018). Impacts of urbanization around Mediterranean cities: Changes in ecosystem service supply. Ecol. Indic..

[60] Jurdi M., Korfali S.I., Karahagopian Y., Davies B.E. (2001). A prototype study for the management of surface water resources, Lebanon. Water Policy.

[61] Hardin P.J., Jensen R.R. (2007). The effect of urban leaf area on summertime urban surface kinetic temperatures: A Terre Haute case study. Urban For. Urban Green..

[62] Zhang J.T., Jiao W.X., Han B.L. (2020). Characteristics of coordination changes and spatial coupling relationship between urbanization and ecosystem services. Acta Ecol. Sin..

[63] Zhao X., Du Y., Li H., Wang W. (2021). Spatio-temporal changes of the coupling relationship between urbanization and ecosystem services in the Middle Yellow River. J. Nat. Resour..

[64] Qiao B., Fang C. (2005). The dynamic coupling model of the harmonious development between urbanization and eco-environment and its application in arid area. Acta Ecol. Sin..

[65] Santos M.F., Martín L.B., García L.M., Aguado M., Benayas J., Montes C. (2013). Unraveling the relationships between ecosystems and human wellbeing in Spain. PLoS ONE.

[66] Liu H., Fang C., Li Y. (2019). The Coupled Human and Natural Cube: A conceptual framework for analyzing urbanization and eco-environment interactions. Acta Geogr. Sin..

[67] Fang C., Cui X., Liang L. (2019). Theoretical analysis of urbanization and eco-environment coupling coil and coupler control. Acta Geogr. Sin..

[68] Zhao A., Li Y., Wei H., Chen X. (2012). Study on coupling coordination development between urbanization and urban eco-environment of Xi’an city. Res. Soil Water Conserv..

[69] Wang W., Wu T., Li Y., Xie S., Han B., Zheng H., Ouyang Z. (2020). Urbanization impacts on natural habitat and ecosystem services in the Guangdong—Hong Kong—Macao “megacity”. Sustainability.

[70] Tapio P. (2005). Towards a theory of decoupling: Degrees of decoupling in the EU and the case of road traffic in Finland between 1970 and 2001. Transp. Policy.

[71] Li W., Wang Y., Xie S., Cheng X. (2021). Coupling coordination analysis and spatiotemporal heterogeneity between urbanization and ecosystem health in Chongqing municipality, China. Sci. Total Environ..

[72] Qiu J., Liu Y., Yuan L., Chen C., Huang Q. (2021). Research progress and prospect of the interrelationship between ecosystem services and human well-being in the context of coupled human and natural system. Prog. Geogr..

[73] Chen X., de Vries S., Assmuth T., Dick J., Hermans T., Hertel O., Jensen A., Jones L., Kabisch S., Lanki T. (2019). Research challenges for cultural ecosystem services and public health in (peri-) urban environments. Sci. Total Environ..

[74] Bai Y., Zhuang C., Ouyang Z., Zheng H., Jiang B. (2011). Spatial characteristics between biodiversity and ecosystem services in a human-dominated watershed. Ecol. Complex..

[75] Das M., Das A., Pereira P., Mandal A. (2021). Exploring the spatio-temporal dynamics of ecosystem health: A study on a rapidly urbanizing metropolitan area of Lower Gangetic Plain, India. Ecol. Indic..

[76] Yan S.G., Zhang H., Li H.D., Tang H. (2017). Ecosystem service values of the entire land area and ecological redlines in Jiangsu Province. Acta Ecol. Sin..

